# Estimation of HIV incidence in two Brazilian municipalities, 2013

**DOI:** 10.1590/S1518-8787.2016050006310

**Published:** 2016-08-26

**Authors:** Célia Landmann Szwarcwald, Orlando da Costa Ferreira, Ana Maria de Brito, Karin Regina Luhm, Clea Elisa Lopes Ribeiro, Ana Maria Silva, Ana Maria Salustiano Cavalcanti, Tomoko Sasazawa Ito, Sonia Mara Raboni, Paulo Roberto Borges de Souza, Gerson Fernando Mendes Pereira

**Affiliations:** I Laboratório de Informações em Saúde. Instituto de Comunicação e Informação Científica e Tecnológica em Saúde. Fundação Oswaldo Cruz. Rio de Janeiro, RJ, Brasil; II Departamento de Genética. Universidade Federal do Rio de Janeiro. Rio de Janeiro, RJ, Brasil; III Departamento de Saúde Coletiva. Centro de Pesquisas Aggeu Magalhães. Fundação Oswaldo Cruz. Recife, PE, Brasil; IV Departamento de Saúde Comunitária. Setor de Ciências da Saúde. Universidade Federal do Paraná. Curitiba, PR, Brasil; V Secretaria Municipal da Saúde de Curitiba. Curitiba, PR, Brasil; VI Laboratório Municipal de Saúde Pública. Secretaria Municipal de Saúde de Recife. Recife, PE, Brasil; VII Laboratório Central de Saúde Pública. Secretaria Estadual de Saúde de Recife. Recife, PE, Brasil; VIII Prefeitura Municipal de Curitiba. Curitiba, PR, Brasil; IX Universidade Federal do Paraná. Curitiba, PR, Brasil; X Departamento de Doenças Sexualmente Transmissíveis, Aids e Hepatites Virais. Ministério da Saúde. Distrito Federal, Brasil

**Keywords:** HIV Infections, diagnosis, AIDS Serodiagnosis, methods, Risk Groups, Acquired Immunodeficiency Syndrome, epidemiology

## Abstract

**OBJECTIVE:**

To estimate HIV incidence in two Brazilian municipalities, Recife and Curitiba, in the year of 2013.

**METHODS:**

The method for estimating incidence was based on primary information, resulting from the Lag-Avidity laboratory test for detection of recent HIV infections, applied in a sample of the cases diagnosed in the two cities in 2013. For the estimation of the HIV incidence for the total population of the cities, the recent infections detected in the research were annualized and weighted by the inverse of the probability of HIV testing in 2013 among the infected and not diagnosed cases. After estimating HIV incidence for the total population, the incidence rates were estimated by sex, age group, and exposure category.

**RESULTS:**

In Recife, 902 individuals aged 13 years and older were diagnosed with HIV infection. From these, 528 were included in the study, and the estimated proportion of recent infections was 13.1%. In Curitiba, 1,013 people aged 13 years and older were diagnosed, 497 participated in the study, and the proportion of recent infections was 10.5%. In Recife, the estimated incidence rate was 53.1/100,000 inhabitants of 13 years and older, while in Curitiba, it was 41.1/100,000, with male-to-female ratio of 3.5 and 2.4, respectively. We observed high rates of HIV incidence among men who have sex with men, of 1.47% in Recife and 0.92% in Curitiba.

**CONCLUSIONS:**

The results obtained in the two cities showed that the group of men who have sex with men are disproportionately subject to a greater risk of new infections, and indicate that strategies to control the spread of the epidemic in this population subgroup are essential and urgent.

## RESUMO

**OBJETIVO:**

Estimar a incidência de HIV em dois municípios brasileiros, Recife e Curitiba, no ano de 2013.

**MÉTODOS:**

O método de estimação da incidência foi baseado em informações primárias, resultantes do ensaio laboratorial *Lag-Avidity* para detecção de infecções recentes do HIV, aplicado em uma amostra dos casos diagnosticados nas duas cidades em 2013. Para a estimação da incidência de HIV para a população total das cidades, as infecções recentes detectadas na pesquisa foram anualizadas e ponderadas pelo inverso da probabilidade de teste de HIV no ano de 2013 entre os casos infectados e não diagnosticados. Após a estimação da incidência de HIV para a população total, foram estimadas as taxas de incidência por sexo, faixa de idade e categoria de exposição.

**RESULTADOS:**

Em Recife, foram diagnosticados 902 indivíduos de 13 anos e mais com infecção de HIV. Desses, 528 foram incluídos no estudo, e a proporção estimada de infecções recentes foi de 13,1%. Em Curitiba, foram diagnosticadas 1.013 pessoas de 13 anos e mais, 497 participaram do estudo, e a proporção de infecções recentes foi de 10,5%. Em Recife, a taxa de incidência estimada foi de 53,1 por 100 mil habitantes de 13 anos e mais, enquanto em Curitiba, de 41,1 por 100 mil, com razão do sexo masculino para o feminino de 3,5 e 2,4, respectivamente. Foram evidenciadas elevadas taxas de incidência de HIV entre homens que fazem sexo com homens, de 1,47% em Recife e 0,92% em Curitiba.

**CONCLUSÕES:**

Os resultados obtidos nas duas cidades mostraram que o grupo dos homens que fazem sexo com homens está desproporcionalmente sujeito ao maior risco de novas infecções, e indicam que estratégias para controle da disseminação da epidemia nesse subgrupo populacional são essenciais e urgentes.

## INTRODUCTION

Epidemiological surveillance information has been considered essential to evaluate the actions of control of the HIV/AIDS epidemic and to subsidize the planning of intervention strategies. In the last two decades, many countries have adopted HIV prevalence as an important indicator of second generation surveillance, which associates the HIV test result with risk behavior^1^.

However, prevalence studies provide the proportion of people infected with HIV, including those with recent infection and with long-term infection. With the expansion of the antiretroviral treatment and, consequently, the survival of individuals infected by HIV^7^, the interpretation of prevalence becomes increasingly difficult, being essential to rely on estimates of HIV incidence for the delineation of the current scenario of the epidemic.

The incidence indicates the degree in which the virus transmission is occurring and the groups under the highest risk of HIV transmission, and allows to identify the emergence of new sub-epidemics of HIV in the general population. Because of its recognized importance, HIV incidence estimates are being incorporated, increasingly, to the surveillance activities in many countries^2^ ^,^ ^8^ ^,^ ^20^ ^,^ ^22^.

Recently, laboratory tests have been developed to estimate the HIV incidence in cross-sectional studies. The algorithms based on laboratory tests make it possible to identify whether the infection is recent or long-term^9^. The main advantage of this type of study is using only one blood sample, collected at a point of time, such as in studies to estimate prevalence, eliminating the follow up of individuals^19^.

In the 2000s, the BED-CEIA test (Calypte HIV-1 BED Incidence Capture EIA) was developed, which is an immunoenzymatic analysis that allows to distinguish cases with recent infection from cases with long-term infection by HIV^21^. The BED-CEIA test was used to estimate the HIV incidence in several countries and different epidemiologic scenarios^5^ ^,^ ^8^ ^,^ ^16^, including between the reported cases of HIV to estimate the HIV incidence in the United States of America (USA)^10^.

However, validation studies have shown, consistently, disparate estimates of HIV incidence when using calculations based on the result of the BED-CEIA test, depending on the adopted parameters and method used for the estimate^17^. Additionally, studies show false-recent results of BED between people with very low CD4 count (< 50/μl) or receiving antiretroviral therapy (ART)^15^.

Another method that allows to distinguish recent infections from long-term infections is based on the IgG avidity test for HIV-1 antigens, first described in 2002. Our methodology is based on the principle that the avidity of the antibodies produced in the early stage of infection is reduced, in opposition to what is observed in long-term infections^24^. Among the advantages of the avidity test, we highlight: the high sensitivity and specificity in detecting recent infections; the simplicity and automatism of the technique; the good performance of the test regardless of HIV subtype; and the fact the avidity index is not affected by the use of ART^6^.

This article aimed to estimate the incidence of HIV in two Brazilian municipalities, Recife and Curitiba, in 2013, by statistical estimation method based on the Lag-Avidity laboratory test results for detection of recent infections, applied to the cases diagnosed with HIV over that year in the two cities.

## METHODS

The employed incidence estimation method used primary information, resulting from laboratory tests to detect recent HIV infections applied in Recife and Curitiba. The two municipalities were chosen for the research because they are located in the Northeast and South, respectively, which have different levels of socioeconomic development and different epidemiological situations regarding HIV infection^4^.

The avidity test (Sedia^TM^ HIV-1LAg-Avidity EIA, Sedia Biosciences Corporation, Portland, Oregon, USA) for detection of recent HIV infection was applied in a sample of positive HIV tests, diagnosed in Recife and Curitiba in 2013. The study was done in partnership with the Centers for Disease Control and Prevention (CDC) and was approved by the Ethics Committee of the Oswaldo Cruz Foundation (Protocol 485,175) and by the CDC responsible body (Office of the Associate Director for Science of the Division of Global HIV/AIDS, US Centers for Disease Control and Prevention).

CDC has licensed the sale of the Sedia^TM^ HIV-1LAg-Avidity EIA in the United States only for research use. Samples classified as recent in the Sedia^TM^ test have an average duration of seroconversion of 141 days (95%CI 119–150). In Brazil, the product is still not registered by Anvisa. The CDC gave the necessary tests for the conduction of the project and paid all the costs of imports. Two lots were used during the study (EK0201 and FA3001), both in Curitiba and Recife.

Individuals who carry out HIV testing in the public sector look for two types of health units: the ones that just collect blood and the ones that collect the blood and do the HIV test. In the first group are the basic health units, which collect the blood and routinely send the samples to municipal laboratories for analysis. In the second are the *Centros de Testagem e Aconselhamento* (CTA – Testing and Counseling Centers), the “Get To Know” trailers, the family health units, which perform HIV rapid test, and the public hospitals.

In health units that collect and perform the HIV test, it was necessary to collect venous blood among individuals with positive result to enable the application of the test of recent infection. The samples of HIV-infected individuals were properly labeled, with their identification, and sent to public laboratories previously selected. As in some health units the positive result of HIV is rare or occurs during non-business hours, as in the case of trailers, the blood samples were collected in gel separator tube, centrifuged, and stored in common refrigerator (from 4°C to 8°C). The dispatch to the reference laboratory was done within 24 hours.

The blood samples of HIV-infected individuals were stored in freezer (-20°C) in the public laboratories for further application of the avidity test for detection of recent infections. To include tests conducted in the private sector, we considered the blood samples that had positive result for HIV in selected private laboratories in the two cities. The blood samples were properly transported and stored in the public laboratories previously selected, for application of the recent detection test, concomitantly with the samples from the public sector.

### Information Bank

In each Municipal Secretariat of Health (SMS), we composed an information bank of individuals who performed the HIV test and had positive results, with the following variables: name, city of residence, sex, date of birth, HIV test date, and laboratory test result (recent or long-term infection).

The information bank was related to other information systems of the municipal secretariats, such as the electronic records system in Curitiba and the Laboratory Tests Requisition System (SIREX) in Recife, and the Information System for testing and counseling centers, in both cities, to add sociodemographic and exposure category information, when available.

The individuals were identified by full name, date of birth, and city of residence, to eliminate duplicates. These are found, for example, between individuals that make the HIV test several times a year, or between those who have received positive results for HIV and repeat the test. After the elimination of duplicates and composition of the information bank in the SMS, the individual’s identification variables were replaced with codes for the statistical analysis of the data.

### Data Analysis

In the proposed model to estimate the HIV incidence in the USA^10^, they assumed that the number of new infections has a Poisson distribution with parameter *I*, while the number of recent infections detected by the laboratory test has a Poisson distribution with parameter *R = IP*, where *P* is the probability that a person infected with HIV is detected with recent infection by the biological marker used in a sample of cases diagnosed in a given year. The probability *P* can be written by the product: *P = P*_1_ . *P*_2_ *. P*_3_, being: *P*_1_ the probability of an individual infected with HIV performing his first test in a given year; *P*_2_ the probability of a person diagnosed with HIV in that year having positive result of the recent infection test; and *P*_3_ the probability of conduction of recent infection test among people diagnosed in a given year^10^.

Thus, if *r* is the number of cases with recent infections detected in the sample of HIV-infected cases, after setting the number of recent infections in the window period for the annual number of recent infections by a mathematical correction factor, the estimator (*i*) of *I*, proposed by McWalter and Welte^17^ (2010), is given by:


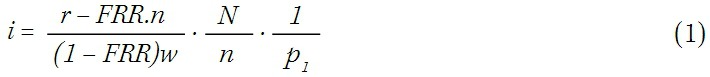


in which,

*N* = number of individuals infected with HIV diagnosed for the first time in 2013 (population);

*n* = number of positive individuals diagnosed in 2013 for which the avidity test was conducted;

*r* = number of recent infections detected in the sample;

*w* = average duration to detect recent infection, expressed as a fraction of the year (equal to 141/365, because the avidity test window period is of 141 days);

*FRR* = false recent rate for the test used (0.2%)^6^;

*p*_1_ = estimator of the probability of a not yet diagnosed positive individual be tested for HIV in a given year.

For the application of the equation (1), the probability *p*_1_ was estimated at 0.44, as the proportion of people infected with HIV who performed HIV test in 2013 between the previously undiagnosed ones. The number of people infected with HIV who performed the HIV test in 2013 (64,070) was obtained by the information of the Control System of Laboratory Tests (SISCEL), and the number of people without previous diagnosis (145,000), by the cascade of continuous care of HIV, Brazil, 2013^4^.

In each city, we compared the distribution of cases classified as recent and long-term by Chi-square test for homogeneity of distributions, according to sex, age group (13-24; 25-34; 35-49; 50+), and exposure category: women; men who have sex with men (MSM); men classified in other exposure categories (heterosexual; injectable drug user; blood; other; not informed).

After the HIV incidence calculation to the total population, we estimated the incidence rates by sex, age group, and exposure category, in each city. To find the number of recent infections in each analysis category, we multiplied the proportion of recent infections in the specific category by the estimated number of people in each category in 2013. The populations by sex and age group were projected for the year 2013 from the information of the Demographic Census, 2010. To calculate the size of the group of men who have sex with men, we multiplied the male population of individuals of 13 years or more estimated for 2013 by 3.5%, estimated proportion of MSM in Brazil^25^.

## RESULTS

In Table 1, we present the results of the laboratory test in Recife and Curitiba. In the first city, the proportion of recent infections was 13.1% and, in the second, 10.5%. Although the punctual value of the proportion of recent infections was higher in Recife, there was no statistically significant difference, with intersection of the confidence intervals.

**Table 1:** Avidity test results. Recife and Curitiba, 2013.

Result	Recife	Curitiba
Number of individuals diagnosed (N)	902	1,013
Number of positive individuals for which the avidity test was conducted (n)	528	497
Number of recent infections detected by the avidity test (r)	69	52
Proportion of recent infections in the sample and 95%CI	13.1% (10.2–15.9)	10,5% (7.8–13.2)
Yearly proportion of recent infections and 95%CI	33.8% (26.4–41.3)	27.2% (20.1–34.1)
Yearly number of recent infections and 95%CI	300 (234–366)	269 (201–338)
Estimated probability that an individual infected with HIV was tested in 2013 (p)^a^	44.0%	44.0%
Estimated number of new cases of HIV and 95%CI	683 (532–833)	612 (456–767)
Estimated population with 13 years or more^b^	1,286,197	1,486,347
Incidence rate (per 100,000 individuals with 13 years or more) and 95%CI	53.1 (41.4–64.8)	41.1 (30.7–51.6)

^a^ Estimated by the cascade of continuous care of HIV, Brazil, 2013^4^.

^b^ Designed by the demographic information disclosed by the DATASUS website.

The application of the equation (1) to the results presented in Table 1 made it possible to estimate the number of new cases of HIV in 2013 in each of the cities. In Recife, the estimated number of new infections was 683, corresponding to an incidence rate of 53.1/100,000 inhabitants of 13 years or more, while in Curitiba, the estimated number of new infections was 612, with an incidence rate of 41.1/100,000 inhabitants of 13 years or more.

Table 2 contains the proportions of recent infections by age group, sex, and exposure category. In both cities, the largest proportion of recent infections is found among young people, under 25 old. In Curitiba, there was a decreasing gradient with increasing age and the differences in the proportions were significant (p < 1.0%). In Recife, the youngest age range also stood out, but the differences were not statistically significant at the 5% level.

**Table 2:** Proportion (%) of recent and long-term infections by sex, age group, and exposure category. Recife and Curitiba, 2013.

Recife		Recent (%)	Long-term (%)	p^a^
Age group (years)	13-24	19.4	80.6	0.230
	25-34	12.1	87.9	
	35-49	11.1	88,9	
	50+	11.8	88.2	
Sex	F	8.9	91.1	0.073
	M	14.5	85.5	
Exposure category	F	8.9	91.1	< 0.001^b^
	MSM	26.0	74.0	
	Men in other exposure categories	9.2	90,8	
Curitiba				
Age group (years)	13-24	18.8	81.2	0.001^b^
	25-34	11.2	88.8	
	35-49	7.5	92.5	
	50+	2.7	97.3	
Sex	F	11.3	88.7	0.313
	M	9.7	90.3	
Exposure category	F	11.3	88.7	0.01^b^
	MSM	15.5	84.5	
	Men in other exposure categories	6.8	93.2	

F: female; M: male; MSM: men who have sex with men

^a^ descriptive level of significance of the Chi-square test for homogeneity of distributions.

^b^ significant at 5% level.

Regarding the comparison by sex, the proportions of recent infections were similar in the two cities (Table 2). Regarding the exposure category, in both cities, the proportions of recent infections were significantly higher among men who have sex with men (MSM), both when compared to women and when compared to other men classified in other exposure categories. The results displayed in Table 2 show that the differences by exposure category were significant at the 1% level, in the two studied cities.

Table 3 shows the incidence rates (per 100,000 inhabitants) estimated by sex, age group, and exposure category. The highest rate was found in the group of 25 to 34 years old, and the lowest, between people with 50 years or more, in both cities. Concerning the analysis by sex, the sex ratio (3.4) in the city of Recife is more pronounced than in Curitiba (2.4), with higher risk among men. However, the results that most stand out in Table 3 are the high rates of HIV incidence in the MSM group. In Recife, the incidence rate of HIV exceeds 1.0%, while in Curitiba, it is close to 1.0% (0.92%). Additionally, we observe that the incidence rates of HIV for females are similar in both cities. The biggest differences are in the incidence rates for males, MSM group, especially in Recife.

**Table 3:** Incidence rates of HIV (per 100,000 inhabitants) according to age group, sex, and exposure category. Recife and Curitiba, 2013.

		Incidence rate of HIV (per 100,000 inhabitants)	

		Recife	Curitiba
Age group (years)	13-24	58.7	60.2
	25-34	78.9	74.1
	35-49	57.9	33.9
	50+	22.7	4.4
Sex	F	25.2	25.1
	M	87.3	59.4
Exposure category	F	25.2	25.1
	MSM	1,469.0	923.7
	Men in other exposure categories	37.2	28.1
Total (13 years or more)		53.1	41.2

F: female; M: male; MSM: men who have sex with men

## DISCUSSION

In Brazil, the serological tests for detecting recent infection have been applied since the late 1990s^19^. However, laboratory tests have never been used in Brazil, previously, to estimate the HIV incidence in the general population.

In this study, the avidity test has been applied in a sample of the HIV cases diagnosed in 2013, in Recife and Curitiba, to investigate the possibility of using laboratory tests to detect recent HIV infections, routinely in Brazil, allowing the monitoring of HIV incidence in the major cities of the Country.

Our methodology was feasible, especially for HIV cases diagnosed in the public sector. Among the individuals who perform the HIV test in units that only collect blood, the samples are sent to the municipal laboratories for analysis, routinely, being possible to perform the test of recent infection for all positive cases. Among the units that collect and perform the HIV test, the planning of venous blood collection among the positive cases was necessary. In Recife, among the cases diagnosed with HIV, the venous collection is routinely performed for the syphilis test. However, in Curitiba, this procedure is no longer being adopted because of the use of rapid tests for syphilis, and it was necessary to use venous blood samples collected for the first CD4 T lymphocyte count. Although all venous blood samples have been scheduled in a maximum period of 15 days from the date of HIV diagnosis, there was a higher number of losses than in Recife and possible delay in the venous blood collection.

Regarding the private sector, the logistics of deploying the laboratory test for detecting recent infection was more complicated. In private laboratories, not all blood samples are tested locally and the confirmatory HIV test is usually performed in another city. To consider individuals diagnosed with HIV in private laboratories, the laboratories that perform, locally, the ELISA tests, needed to store part of the venous blood sample of the positive individuals before the conduction of the confirmatory test. The blood samples stored in the private laboratory were transported to public laboratories for the confirmatory HIV testing and for detecting recent infection among the confirmed cases. The inclusion of private sector cases was made by agreement with selected laboratories in the two cities and only since May 2013. In addition, as we did not consider the positive samples of blood banks and hospitals with less than five positive cases per month, the avidity test has been applied in 58.5% of cases diagnosed in Recife and 49.0% in Curitiba.

In countries with concentrated epidemics, the population-based studies by sampling for estimation of the proportion of recent infections are impractical, both by requiring very large sample sizes and by the difficulties of attracting individuals under greater risk to HIV^14^. These countries have chosen to estimate HIV incidence based on reported cases of HIV, considering all individuals infected with HIV diagnosed in a given year as population elements, while those detected as suffering recent infections as sample persons. The selection probabilities are estimated by the probabilities of individuals infected with HIV, not diagnosed earlier, be tested in a given year. Recent infections detected in the research are weighted by the inverse of the probabilities of selection (expansion factors) to produce the number of incident cases in the year^8^ ^,^ ^10^.

In this study, for the application of the method in the two Brazilian cities, we used McWalter and Welte^17^ calculation, the most recommended among the proposed mathematical formulas, because it is a simplified and updated version and for not presenting statistically significant differences when compared to the other estimates. In both cities, the annualized number of recent infections was weighted by the inverse of the proportion of HIV-infected cases in Brazil, which were tested in 2013 and had no previous diagnosis^4^. Because of the unavailability of specific estimates of this proportion for Recife and Curitiba, we used the weighting factor estimated for all Brazil in the two cities. This was the main limitation of the study, since large differences in the weighting factor may lead to relevant changes in the incidence estimates.

The results were consistent with previous Brazilian studies. In Curitiba, the incidence rate of HIV is on the level of 40/100,000 individuals with 13 years or more, while in Recife, it exceeded 50/100,000. According to data of the latest Epidemiological Bulletin, Recife occupies the eighth place among Brazilian capitals because of the high detection and mortality rates, while Curitiba occupies the 16^th^ position^4^.

Both in Curitiba and in Recife, the highest prevalences of recent infection have occurred in young people, with the highest incidence rate in the group of 25 to 34 years old. In both cities, the incidence rates were higher among men, but the sex ratio was much more pronounced in Recife, with 3.4 new infections among men for every new infection among women. In a previous study carried out in Recife, the proportion of recent infections for males was 2.4 times greater than for females^11^, equal value to the one found for Curitiba in this study. The increase is probably explained by the increasing number of incident cases among men who have sex with men, which accounted for 43.5% of recent infections, in Recife, and 36.8%, in Curitiba.

In fact, the results that most called the attention in the study were the high incidence rates of HIV in the MSM group. In Curitiba, the incidence rate among MSM is close to 1.0%, and, in Recife, it exceeded this value. It is noteworthy that all male cases without reported exposure category were classified as “male in other exposure category”, i.e., the incidence rate among MSM may be even greater than the estimated here.

Despite earlier concerns about an increase in heterosexual cases and “feminization” of the epidemic, the findings of this study indicate a predominance of new infections among MSM, corroborating evidence of a resurgence of the epidemic in this population group in other countries^3^ ^,^ ^23^. A research conducted incidence 10 Brazilian cities, using Respondent Driven Sampling as sampling method, showed prevalence of HIV of 14.2% among MSM^11^, approximately 30 times greater than the prevalence of HIV in the heterosexual male population. In an investigation of the composition of recent infections by exposure category, held in testing centers in Rio de Janeiro in 2004-2005, the estimated incidence among MSM was 11 times greater than among heterosexual men^5^.

Despite the evidence of increased risk among MSM, the periodic test coverage for HIV in this population subgroup is still low and insufficient to ensure the early detection and immediate treatment^13^. The already widely recognized benefits of early introduction of antiretroviral therapy^18^ seem to be neglected by the stigma and fear of being positive^16^.

In sum, the results obtained in the two cities showed that the MSM group is disproportionately subject to greater risk of new infections, and indicate that strategies to control the spread of the epidemic in this population subgroup are essential and urgent.
